# Wall enhancement predictive of abnormal hemodynamics and ischemia in vertebrobasilar non-saccular aneurysms: a pilot study

**DOI:** 10.3389/fneur.2023.1108904

**Published:** 2023-06-02

**Authors:** Yeqing Jiang, Liang Ge, Gang Lu, Hailin Wan, Qi Chen, Rong Zou, Xiaochang Leng, Jianping Xiang, Xiaolong Zhang

**Affiliations:** ^1^Huashan Hospital, Fudan University, Shanghai, China; ^2^ArteryFlow Technology Co., Ltd, Hangzhou, China

**Keywords:** computed fluid dynamics, wall enhancement, vertebrobasilar fusiform aneurysm, high-resolution MRI, oscillatory shear index

## Abstract

**Objective:**

To analyze how wall enhancement affects hemodynamics and cerebral ischemic risk factors in vertebrobasilar non-saccular intracranial aneurysms (VBNIAs).

**Materials and methods:**

Ten consecutive non-saccular aneurysms were collected, including three transitional vertebrobasilar dolichoectasia (TVBD). A wall enhancement model was quantitatively constructed to analyze how wall enhancement interacts with hemodynamics and cerebral ischemic factors.

**Results:**

Enhanced area revealed low wall shear stress (WSS) and wall shear stress gradient (WSSG), with high oscillatory shear index (OSI), relative residence time (RRT), and gradient oscillatory number (GON) while the vortex and slow flow region in fusiform aneurysms are similar to TVBD fusiform aneurysms. With low OSI, high RRT and similar GON in the dilated segment, the enhanced area still manifests low WSS and WSSG in the slow flow area with no vortex. In fusiform aneurysms, wall enhancement was negatively correlated with WSS (except for case 7^1^, all *p* values  < 0.05, *r* = −0.52 ~ −0.95), while wall enhancement was positively correlated with OSI (except for case 5, all *p* values < 0.05, *r* = 0.50 ~ 0.83). For the 10 fusiform aneurysms, wall enhancement is significantly positively correlated with OSI (*p* = 0.0002, *r* = 0.75) and slightly negatively correlated with WSS (*p* = 0.196, *r* = −0.30) throughout the dataset. Aneurysm length, width, low wall shear stress area (LSA), high OSI, low flow volume (LFV), RRT, and high aneurysm-to-pituitary stalk contrast ratio (CRstalk) area plus proportion may be predictive of cerebral ischemia.

**Conclusion:**

A wall enhancement quantitative model was established for vertebrobasilar non-saccular aneurysms. Low WSS was negatively correlated with wall enhancement, while high OSI was positively correlated with wall enhancement. Fusiform aneurysm hemodynamics in TVBD are similar to simple fusiform aneurysms. Cerebral ischemia risk appears to be correlated with large size, high OSI, LSA, and RRT, LFV, and wall enhancement.

## Introduction

Vertebrobasilar non-saccular intracranial aneurysms (VBNIAs), including fusiform aneurysms (FAs) and transitional vertebrobasilar dolichoectasia (TVBD), have a poor natural history. Longitudinal follow-up studies show an annual growth rate of 6%, a 2% annual rupture rate, with ischemic stroke occurring in 6% of such cases. Annual mortality is high at 13% (1, 2) with recurrent brainstem infarction (3). Size and irregularity can lead to aneurysm instability (4). Aneurysm possibly resulting in growth, hemorrhage, parenchymal hemorrhage and/or mass effect, including direct compression of the third ventricle floor or “water hammer” resulting in hydrocephalus (3). Endovascular treatment can be beneficial but carries with it a significant risk of brainstem infarction (as high as 22%) for TVBD (5). Therefore, the risks of treatment vs. monitoring should be assessed carefully.

Intracranial Doppler studies have shown that the cause of cerebral ischemic stroke in TVBD patients may be reduced forward blood flow with decreased mean systolic velocity promoting occlusion of perforators (6, 7). Hemodynamics have been widely studied to predict intracranial aneurysm growth and rupture (8). The hemodynamic mechanism of cerebral ischemic events secondary to TVBD and FAs are unclear. Recently, high-resolution magnetic resonance imaging (HR-MRI) studies have shown that wall enhancement is an iconic indicator of aneurysm instability including growth, rupture, or symptomatic issues (9). However, no study has examined the correlation between wall enhancement of vertebrobasilar non-saccular aneurysm and intraluminal hemodynamics. Therefore, this study aims to investigate the correlation between the enhancement characteristics of aneurysm walls and intraluminal hemodynamics and their relationship with cerebral ischemic events, to better understand the mechanism leading to the instability of such lesions.

## Materials and methods

### Population

With the consent of the ethics committee of Huashan Hospital affiliated to Fudan University, ten consecutive patients comprising 3 TVBDs and 7 FAs completing both HR-MRI and digital subtracted angiography (DSA) were collected for this study. Exclusion criteria: patients with poor quality HR-MRI images due to motion artifacts, unobservable aneurysm wall enhancement due to slow flow or with other intracranial vascular diseases (such as saccular aneurysm, arteriosclerosis stenosis, arteriovenous malformations). Aneurysm-related cerebral ischemia was defined as vertebrobasilar infarctions or transient ischemic attack (TIA) history (Table 1). Vertebrobasilar non-saccular intracranial aneurysms (VBNIAs) were further categorized into 3 previously described subtypes (fusiform, dolichoectasia, and transitional) (2).

**Table 1:** Clinical information and patient symptoms.

Case	Initial presentation	CI/TIA	TVBD
1	Dizziness, vomiting	TIA	0
2	Transient dizziness, vomiting	TIA	0
3	Severe headache, single episode	/	0
4	Incidental	/	0
5	Incidental	/	0
6	Slow reaction and memory decline	CI	1
7	Transient slurred speech and facial paralysis	CI	1
8	Incidental	/	0
9	Unsteady gait, lethargy, difficulty speaking	CI	1
10	Transient limb numbness	CI	0

CI, cerebral infarction; TIA, transient ischemic attack.

### High-resolution MRI scans

**Figure 1:**
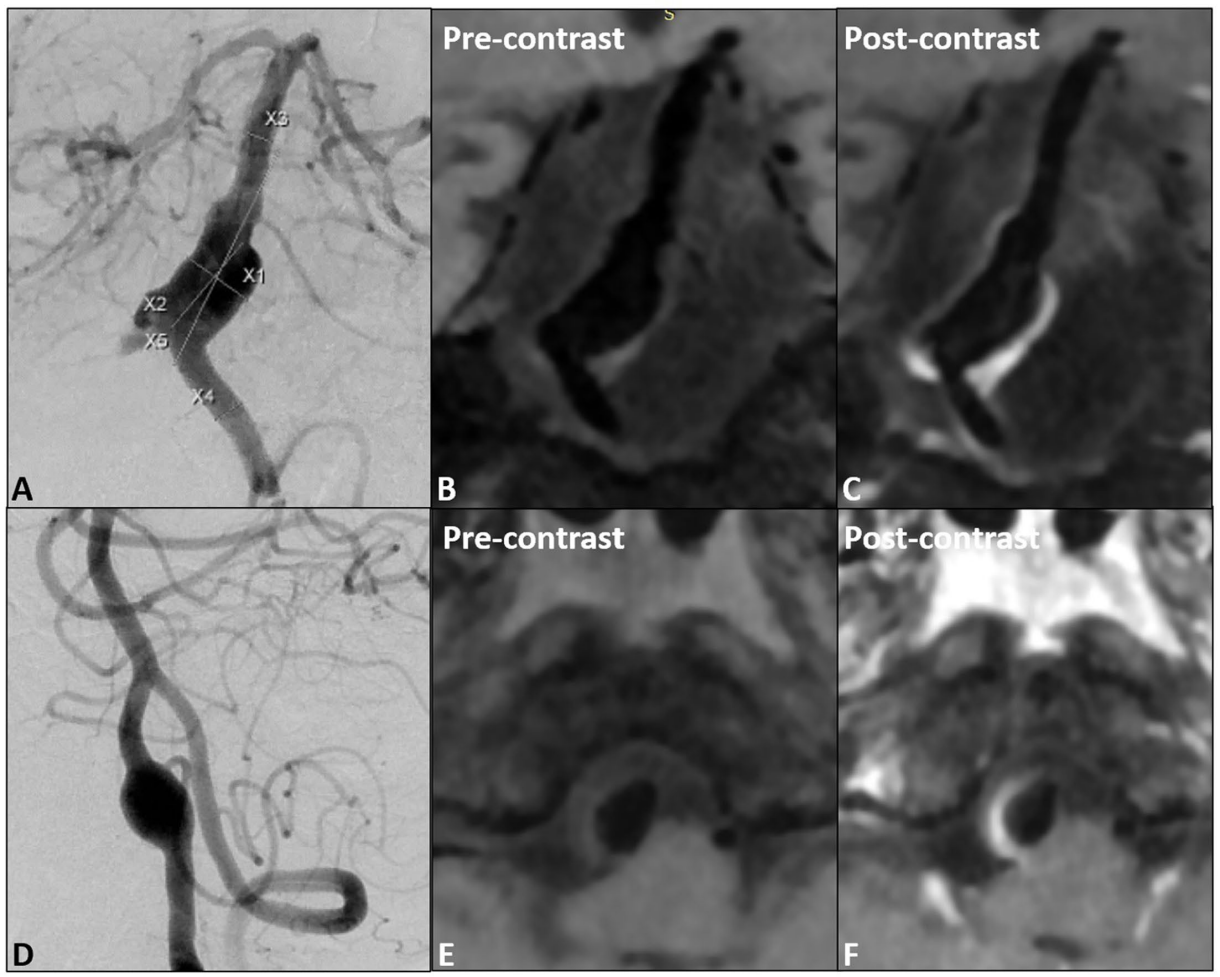
**(A)** The proximal fusiform aneurysm of the basilar artery, **(B,C)** pre- and post- wall enhancement; **(D)** one vertebral fusiform aneurysm, **(E,F)** pre- and post-wall enhancement.

All patients were scanned with 3.0 T GE 750 scanner, 32-channel head coil, TOF-MRA, T1 3D CUBE pre- and post-enhancement (FOV 20 × 19 × 16 cm^3^, repetition time/echo time 600/minimum, matrix 288 x 288x160 interpolation to 512 x 512x320, spatial resolution rate 0.4 × 0.4×0.5 mm^3^). Gadolinium (Gadovist, Bayer Schering Pharma, Berlin, Germany) was injected intravenously at 0.1 mmol/kg. The enlarged images of the typical basilar and vertebral fusiform aneurysm cases pre- and post-enhancement are shown in Figure 1. The specific scanning parameters are available in Table 2.

**Table 2:** 3D-CUBE T1WI plain scan and enhancement, 3D-TOF-MRA specific parameters.

	3D T1WI FSE	3D-TOF
Coil-channel	32	32
TR (ms)	600	25
TE (ms)	Minimum	5.7
FOV (cm)	20*19	22*20
Matrix	288*288	320*256
Spatial resolution rate (mm^3^)	0.4*0.4	0.42*0.42
Thickness (mm)	0.5	0.6
Bandwidth (kHz)	62.5	20.83
Echo chain length	24	–
Velocity encoding (cm/s)	–	–
Acquisition time	4 min 16 s	3 min 22 s

### Model reconstruction

The lumen was reconstructed using ITK-SNAP (version 3.6.0-RC1; http://www.itksnap.org) for enhanced HR-MRI sequences while 3D rotated DSA images were segmented via MIMICS (Mimics 10.0; Materialise, Leuven, Vlaams-Brabant, Belgium) to obtain a 3D lumen model, both exported as STL format files. DSA acquisition model via matching HR-MRI model was used for wall enhancement model reconstruction via image intensity reading. Detailed model reconstruction strategies are shown in the Supplementary material.

### Hemodynamic simulation

In this study, a fully unstructured mesh with a 3-layer boundary was generated using the Star-CCM+ meshing tool (CD-Adapco, Melville, NY, United States). These meshes are used to solve the Navier–Stokes equations governing flow via the Star-CCM+ CFD solver, with a second order accuracy scheme, where blood is modeled as an incompressible laminar Newtonian fluid with a density of 1,056 kg/m^3^ and a viscosity of 0.0035 kg/ms. A no-slip boundary condition is assumed for the vessel wall, and a velocity boundary condition for the inlet. The average volumetric inflow rate was 1.3 mL/s. The flow boundary condition is set at each outlet, based on the principle of minimum energy, to ensure that the flow rate of each outlet is proportional to the cube of its equivalent diameter (10). The pulsatile flow simulation is run for three cardiac cycles to ensure that numeric stability has been achieved, with the result of the third cardiac cycle used for post-processing. Related hemodynamic simulation details and parameters are available in the Supplementary material.

### Statistical analysis

For the quantitative analysis, all measured image intensities and hemodynamic parameters are derived from the same coordinates of the point. Since the measurement point of the image intensity is in the middle layer of the vessel wall and the measurement point of the hemodynamic wall parameters is in the inner wall of the vessel, a script was used to project the image intensity measured on the middle layer of the vessel wall to the closest point on the lumen surface, generating a vtp file containing image intensity, WSS and OSI. The vtp file was imported into Paraview, the lesion area selected and extracted, and the image intensity exported, with WSS and OSI corresponding to each point. Each case is divided into 20 uniform classes according to the distribution interval of its hemodynamic parameters (WSS and OSI), while the width of each class is 5% of the maximum value of the hemodynamic parameters (11). The average value of the hemodynamic parameters and their corresponding image intensities were calculated for all points of each level, and the averaged hemodynamic parameters and image intensities were obtained for 20 groups. This was graphed with the hemodynamic parameters as the X-axis, and the image intensity as a scatter plot for the Y-axis. SPSS (version 21.0, IBM Corp, Armonk, NY) was used for numerical analysis of the results, and the scatter plot underwent linear simulation. The correlation coefficient r and the determination coefficient R2 between the hemodynamic parameters and image intensity for each group of cases were calculated and obtained. The Mann–Whitney *U* test was used to analyze the differences of clinical, morphological, hemodynamic and aneurysm wall enhancement parameters between cerebral ischemia and non-cerebral ischemia groups. *p* < 0.05 was considered statistically significant.

## Results

### Clinical information

Ten patients with 7 fusiform aneurysms and 3 TVBD were enrolled, comprised of 7 males and 3 females, with a median age of 55.50 ± 7.01 (48–65 years old). The ischemic symptom group contained six patients, 4 with a history of cerebral infarction and 2 with TIAs.

### Correlation analysis of aneurysm wall enhancement and intraluminal fluid dynamics

**Figure 2:**
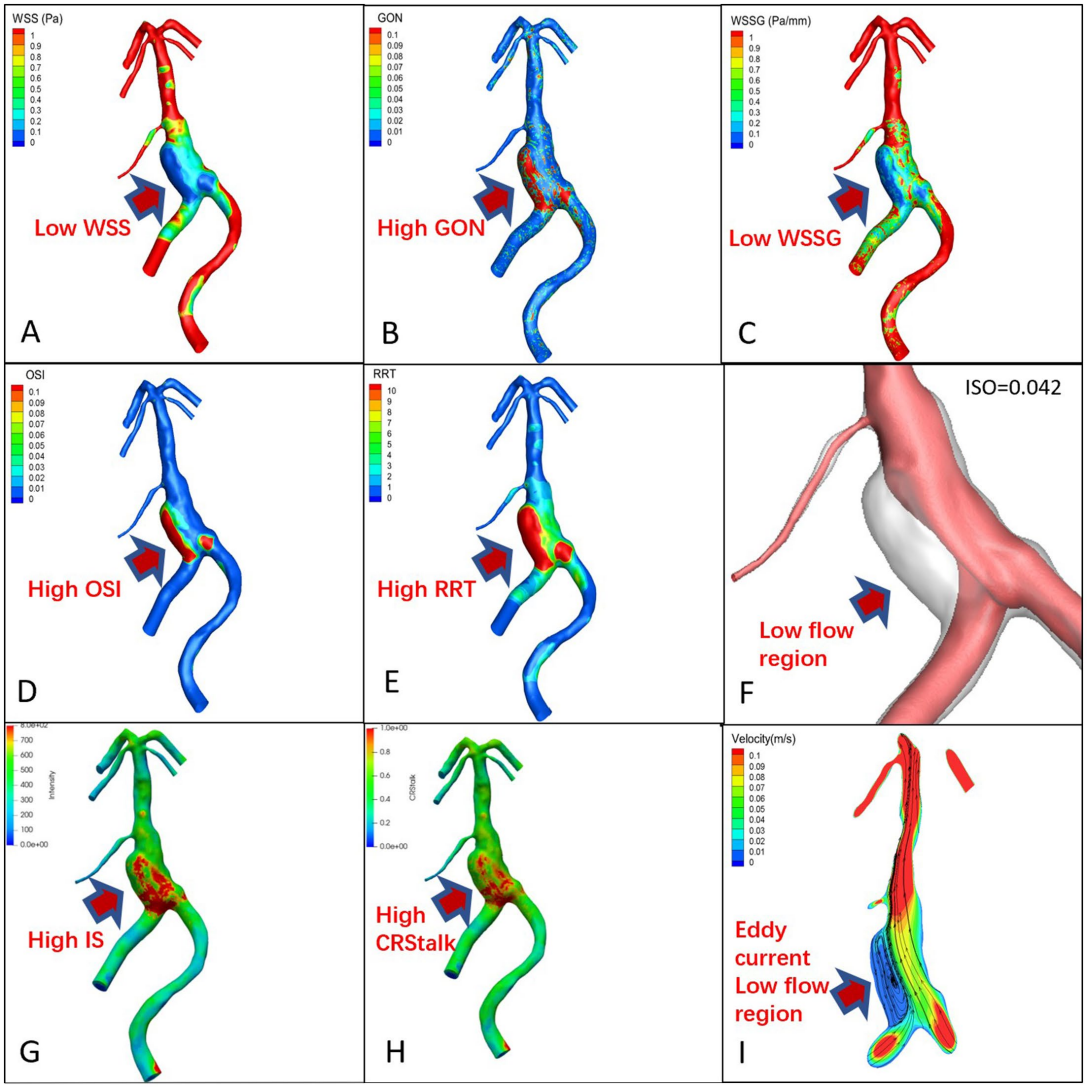
Proximal basilar fusiform aneurysm cloud map, **(A)**: WSS, **(B)**: GON, **(C)**: WWSG, **(D)**: OSI, **(E)**: RRT, **(F)**: Low flow region (isovelocity = 0.042 m/s), **(G)**: aneurysm wall intensity, **(H)**: CRstalk, and **(I)**: eddy current map.

**Figure 3:**
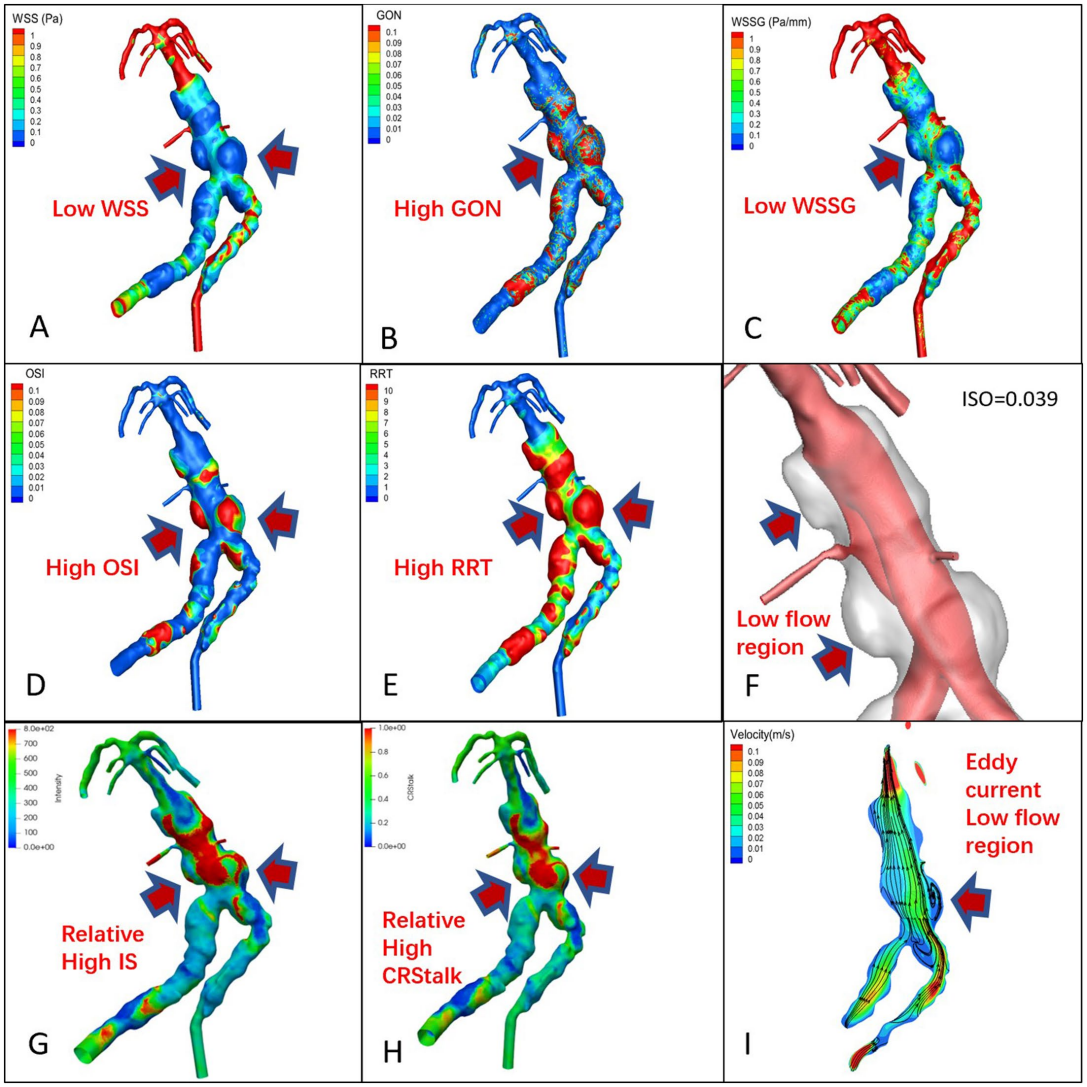
TVBD cloud map, **(A)**: WSS, **(B)**: GON, **(C)**: WWSG, **(D)**: OSI, **(E)**: RRT, **(F)**: Low flow region (isovelocity = 0.039 m/s), **(G)**: aneurysm wall intensity, **(H)**: CRstalk, and **(I)**: eddy current map.

The signal intensity (SI) distribution, aneurysm wall enhancement and CRstalk cloud image of 10 aneurysm wall models were calculated and visualized (Cases 1 and 6; Figures 2G, 3G and 2H, 3H). Image intensity distribution was uneven in all patient. WSS, OSI, WSSG, RRT, GON, LFV, and streamline diagrams were obtained for the 10 aneurysm models (Cases 1 and 6; Figures 2, 3).

#### Qualitative analysis

In the 7 pure fusiform aneurysms, the enhanced area of the vessel wall exhibited low WSS and WSSG, while the low velocity area experienced high OSI, RRT, and GON with obvious eddy currents (Figure 2). In the 3 cases of TVBD, the hemodynamic performance of the enhanced area of the fusiform aneurysm vessel wall showed a similar pattern to that of the 7 pure fusiform aneurysm cases (Figure 3). The enhanced area of the vessel wall of the non-fusiform dilated and tortuous area maintained a low flow velocity with simultaneous low WSS, WSSG and OSI, high RRT and normal GON, with no obvious vortex (Figure 3).

#### Quantitative analysis

**Figure 4:**
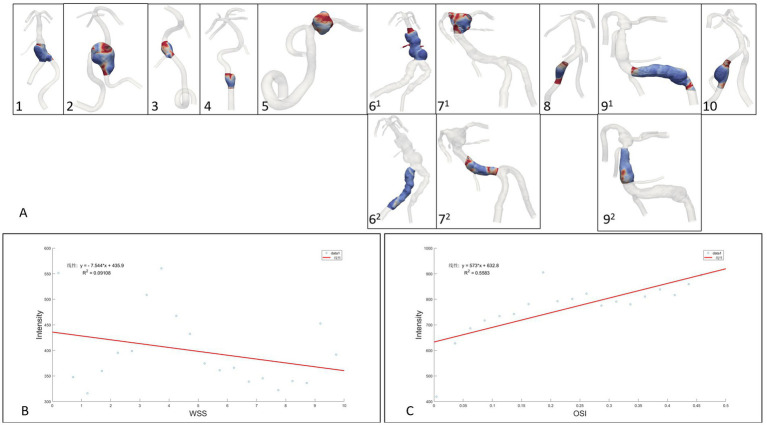
**(A)**: Extraction range of the region of interest enhanced by the aneurysm wall, **(B,C)**: Linear regression analysis of fusiform aneurysm overall data points for wall enhancement, WSS and OSI.

The 10 fusiform aneurysm regions were extracted separately, while the non-aneurysmal dilated lesions of the TVBD were extracted simultaneously (Figure 3). The range of data points extracted for each case ranged from 6,401 to 82,877 (see Table 3 for details). For the 10 fusiform aneurysms, enhancement of the aneurysm wall was negatively correlated with WSS (all *p* values <0.05, *r* = −0.52 ~ −0.95), with the exception of the fusiform aneurysm at the top of the basilar artery in case 7. Enhancement was positively correlated with OSI as a whole (all *p* values <0.05, *r* = 0.50–0.83, Figure 4 and Table 3), except for case 5.

**Table 3:** Number of data points extracted from 10 cases and linear correlation between aneurysm wall signal intensity (SI), WSS, and OSI, along with the correlation coefficient *r* and determination coefficient R2 of the regression equation.

Case	Data points in the lesion area	R2 (WSS vs.SI)	r (WSS vs.SI)	*p*-value	R2 (OSI vs.SI)	*r* (OSI vs.SI)	*p*-value
1	7,742	0.2723	−0.521824	0.0143	0.4511	0.67164	0.013
2	23,746	0.5199	−0.721041	0.0003	0.6926	0.832226	<0.0001
3	6,401	0.7265	−0.85235	<0.0001	0.3323	0.576455	0.0078
4	16,143	0.496	−0.704273	<0.0001	0.346	0.588218	0.0268
5	14,399	0.911	−0.954463	<0.0001	0.06415	−0.253279	0.2813
6^1^	21,351	0.4853	−0.696635	0.0006	0.5384	0.733757	0.0002
6^2^	60,883	0.6865	0.828553	<0.0001	0.4398	0.663174	0.0014
7^1^	35,282	0.008728	0.093424	0.6952	0.5309	0.728629	0.0003
7^2^	37,941	0.0757	−0.275136	0.2404	0.5767	0.759408	0.0001
8	82,877	0.6766	−0.822557	<0.0001	0.5419	0.736139	0.0002
9^1^	67,542	0.6318	−0.794858	<0.0001	0.6786	0.823772	<0.0001
9^2^	26,514	0.1499	0.387169	0.0917	0.6786	0.823772	<0.0001
10	39,041	0.5796	−0.761315	0.0001	0.2453	0.495278	0.0264

For three non-fusiform aneurysm sites, wall enhancement was all significantly positively correlated with OSI (both *p* < 0.05, *r* = 0.66–0.82), while aneurysm wall enhancement significantly and positively correlated with WSS only in case 6^2^ (*p* < 0.0001, *r* = 0.83，Figure 4 and Table 3). Linear regression analysis performed on all data points of aneurysm enhancement extracted from the 10 fusiform aneurysm sites with corresponding WSS and OSI, determined that SI was slightly negatively correlated with WSS (*p* = 0.196, *r* = −0.30), with a significant positive correlation for OSI (*p* = 0.0002, *r* = 0.75). Comparing all data points of aneurysm enhancement extracted from 3 cases of non-fusiform aneurysms with the corresponding WSS and OSI, determined that SI was positively correlated with WSS (*p* = 0.0550, *r* = 0.4354), and SI was positively correlated with OSI (*p* = 0.103, *r* = 0.3755).

#### Wall enhancement and hemodynamics predictive of ischemic events

By calculating the volume of the area 10, 20, 30, and 40% lower than the normal vascular flow rate, and obtaining those volume ratios, the 30% threshold of LFV can clearly differentiated symptomatic cerebral ischemic event (*p* = 0.038).

For prediction of ischemic events and analysis of clinical, morphological and hydrodynamic risk factors, the results of the Mann–Whitney *U* test show that aneurysm length, width, LSA, high OSI, LFV (30%), RRT, and high CRstalk area as well as proportion were statistically different (Table 4).

**Table 4:** Risk factors for clinical, morphological and hemodynamic parameters of cerebral ischemic events.

Parameters	Ischemic (6)	Non-ischemic (4)	*p*-value
Age	55.33 ± 5.75	55.75 ± 9.61	0.876
Gender (M)	5 (83.3)	2 (50)	0.5
HTN	3 (50)	1 (25)	0.571
DM	1 (16.7)	0	1
AN maximum Diameter	21.07 ± 14.42	8.51 ± 1.64	0.019
AN width	11.30 ± 2.71	6.59 ± 1.12	0.010
WR-value	2.57 ± 0.81	2.02 ± 0.48	0.257
Branch-involved	5 (83.3)	1 (25)	0.19
TVBD	3 (50)	0	0.2
TAWSS	0.47 ± 0.24	0.67 ± 0.19	0.257
LSA	0.50 ± 0.29	0.90 ± 0.09	0.038
hOSI	0.01 ± 0.01	0.001 ± 0.002	0.038
OSI	0.04 ± 0.02	0.02 ± 0.01	0.114
LFV (30%)	0.59 ± 0.14	0.35 ± 0.16	0.038
RRT	10.77 ± 5.67	3.78 ± 1.62	0.038
WSSG	1.43 ± 1.17	2.16 ± 1.49	0.476
GON	0.07 ± 0.40	0.04 ± 0.02	0.257
SI	499.26 ± 99.16	450.12 ± 65.57	0.352
hCRstalk (>1)	89.21 ± 53.44	4.94 ± 4.49	0.011
hCRstalk% (>1)	0.11 ± 0.59	0.02 ± 0.02	0.019
CRstalk	0.57 ± 0.13	0.45 ± 0.10	0.257

AN, aneurysm; WR, width ratio; TVBD, transitional vertebrobasilar artery dilatation; TAWSS, time averaged wall shear stress; LSA, low wall shear stress area; OSI, oscillatory shear index; LFV, Low flow volume; RRT, relative blood retention time; WSSG, wall shear stress gradient; GON, gradient oscillatory number; SI, signal intensity.

## Discussion

Aneurysm morphological, hemodynamic and biological changes interact with each other. The hemodynamic changes in the sac have a clear impact on the aneurysm wall. Meng et al. (8) summarized the relationship between aneurysm morphological changes, hemodynamics and biological signals, and concluded that variation in aneurysm morphology immediately cause intraluminal hydrodynamic change. These interact with the aneurysm wall resulting in pathological changes, with such remodeling and degradation of the tube wall leading to morphological changes in the aneurysm sac. Meanwhile aneurysm wall enhancement is associated with atherosclerotic lesions, inflammatory cell infiltration, intraluminal thrombus, and wall vasa vasorum (12). Sricharan et al. (13) investigated the relationship between wall enhancement, risk metrics and aneurysmal flow concluding that the rupture resemblance score (RRS) correlates with wall enhancement.

The study of some representative hemodynamic parameters can help to understand the mechanism of the interaction mentioned above. A thin aneurysm wall is positively correlated with high WSS (11). Swiatek et al. (14) reported that aneurysm wall enhancement was associated with decreased anti-inflammatory cytokine interleukin-10 and unstable morphological parameters, further confirming that inflammatory cell infiltration is one of the pathological mechanisms of wall enhancement. Abnormally high WSS can lead to remodeling of the vessel wall resulting in aneurysms, while abnormally high or low WSS mediates the development of aneurysms (8) through: 1. Eendothelial cell-mediated pathways, through which high WSS causes parietal cells to produce extracellular morphological changes caused by matrix proteases, extracellular matrix degradation and necrosis; 2. the inflammatory cell-mediated pathway causes endothelial cells to become pro-inflammatory cells and prolong their residence time due to low WSS, producing extracellular matrix proteases, leading to the degradation and necrosis of the extracellular matrix, and causing the development of aneurysms. These two pathways can coexist. Treatment is needed to ameliorate such dangerous hemodynamic environments.

Wall enhancement can be divided into local and circumferential aneurysmal wall enhancement (15), necessitating an analysis based upon local parameters of the aneurysm wall. In this study, WSS in the fusiform aneurysm area was significantly negatively correlated with aneurysm wall enhancement (except for case 7^1^), and OSI was significantly positively correlated with aneurysm wall enhancement (except case 5), while the fusiform aneurysm lumen area showed low WSS and high OSI, consistent with previous hemodynamic patterns high rupture risk areas (16). At the same time, the obvious enhancement of the dilated and tortuous vessel wall, representing a significant inflammatory response, is consistent with the low WSS in the inflammatory cell-mediated pathway. Hadad et al. (17) reported 23 cases of saccular aneurysm wall enhancement with low WSS, and confirmed that the hemodynamic environment and enhancement patterns varied in different parts of the aneurysm, with high WSS and high WSSG near the inflow tract of the aneurysm neck, while sites in the body and dome further from the inflow tract were associated with heterogeneous low WSS and enhancement. Sricharan et al. (13) detected a weak relationship between normalized WSS and wall enhancement, and no correlation between OSI and wall enhancement, which may have been affected by their statistical method. As the hemodynamic parameters were unevenly distributed, ranked array would have been helpful to reflect the relationship between the parameters (11).

In case 7^1^, an apical fusiform aneurysm may have been affected by the hemodynamics of multiple large branches, so that the wall enhancement and wall shear stress were not closely related. In case 5, the OSI and SI were slightly negatively correlated. A possible reason for this contrary result was that the regular shape, small aneurysm angle and lack of an involved branch created a simple flow pattern which produced only tiny scattered high OSI areas. With such limited high OSI areas, the average OSI was significantly lower than in other cases, hence the relationship between OSI and SI was weakened.

Vertebrobasilar fusiform aneurysms and VBDs have a poor natural history, especially TVBD (2), due to extensive involvement of the parent artery. Transitional VBDs have risk factors of both fusiform aneurysms and VBDs. Fusiform aneurysms have a larger degree and extent of wall enhancement than saccular aneurysms (18). The hemodynamic and aneurysm wall enhancement patterns of transitional VBDs are more complicated than those of fusiform aneurysms. This study is the first to conclude that the hemodynamic patterns of fusiform aneurysms in a transitional VBD is similar to that of simple fusiform aneurysms. He et al. (19) reported 19 cases of VBD successfully treated with large woven stents with or without coils, and the prognoses at follow-up were satisfactory. Flow diversion devices are a promising alternative after considering the ischemic risk (20). For non-fusiform dilated and tortuous sites, wall enhancement was slightly positively correlated with low OSI and WSS, but without statistical difference. One possible explanation is that the dilated and tortuous segment markedly enhanced wall is located in the pathway mediated by low WSS inflammatory cells.

Transcranial Doppler studies have shown that VBD may cause ischemic stroke due to decreased antegrade blood flow, decreased mean systolic velocity, and bidirectional or retrograde blood flow (6). Endothelial damage associated with marked elongation and angulation of the VBD along with hemodynamic changes can also lead to secondary atherosclerotic plaques (7), predisposing arterial branch inlets to deformation, while reduced blood flow in perforators can lead to infarction. In this dataset, all TVBD patients had cerebral infarction, a perforation event, which may be related to long blood retention time, significantly low flow volume, eddy current and extensive vessel wall inflammation. These risk factors are likely to induce thrombosis, leading to perforator occlusion. At the same time, high OSI and low wall shear stress can further aggravate abnormal vascular remodeling, while abnormally dilated blood vessels will further aggravate abnormal hemodynamics.

### Limitations

The sample size is relatively small, due to the time-consuming nature of current simulation analysis of non-saccular aneurysms. Through these cases, we aim to gradually establish a rapid analysis process and tools which can be used in larger sample sizes in the future. This study serves as a preliminary exploration. Considering the extent of damage of the intima of TVBDs and fusiform aneurysms, multiple tiny intimal flaps cannot be simulated, which may affect the hemodynamic results. Analysis of aneurysm wall thickness was not performed due to magnetic resonance resolution limitations. The lack of motion sensitized driven equilibrium (MSDE) and delay alternating with nutation for tailored excitation (DANTE) may affect the wall enhancement results (21). Therefore, patient with obvious mismatches between the reconstructed models of HR-MRI and DSA due to slow blood flow in the aneurysm sac were excluded (22).

## Conclusion

In fusiform aneurysms, low WSS is negatively correlated with wall enhancement, and high OSI is positively correlated with wall enhancement. Transitional VBDs have a wide range of wall enhancement and complex hemodynamic patterns. The pattern of blood flow in fusiform aneurysms with TVBD was similar to that of simple fusiform aneurysms. Cerebral ischemic risk might be associated with large aneurysms, high OSI, LSA, RRT, LFV, and areas of high enhancement. Better understanding of wall enhancement can aid in the detection, development and prognosis of aneurysms.

## Data availability statement

The raw data supporting the conclusions of this article will be made available by the authors, without undue reservation.

## Ethics statement

The studies involving human participants were reviewed and approved by the Institutional Review Board of Huashan Hospital affiliated to Fudan University. The ethics committee waived the requirement of written informed consent for participation.

## Author contributions

XZ and JX conceived and designed the research. YJ acquired the data. YJ, LG, GL, QC, XL, and RZ analyzed and interpreted the data. YJ, QC, RZ and HW performed the statistical analysis. XZ and JX handled the funding and supervision. YJ drafted the manuscript. All authors made critical revisions to the manuscript for important intellectual content and reviewed the final version of the manuscript.

## Funding

This work was supported by the National Nature Science Foundation of China (Grant no. 81771242) and Hangzhou Leading Innovation and Entrepreneurship Team Project. Team name: Intelligent diagnosis and treatment system for cardiovascular and cerebrovascular diseases (TD2022007).

## Conflict of interest

QC, RZ, XL, and JX were employed by ArteryFlow Technology Co., Ltd.

The remaining authors declare that the research was conducted in the absence of any commercial or financial relationships that could be construed as a potential conflict of interest.

## Publisher’s note

All claims expressed in this article are solely those of the authors and do not necessarily represent those of their affiliated organizations, or those of the publisher, the editors and the reviewers. Any product that may be evaluated in this article, or claim that may be made by its manufacturer, is not guaranteed or endorsed by the publisher.
