# Supplementary material for: Wall enhancement predictive of abnormal hemodynamics and ischemia in vertebrobasilar non-saccular aneurysms: a pilot study
Source: Front Neurol. 2023 Jun 2;14:1108904. doi: 10.3389/fneur.2023.1108904 (PMC10272805; doi:10.3389/fneur.2023.1108904)
Supplement: Supplementary file 1 [file Data_Sheet_1.docx]

**Model reconstruction**

**Lumen model reconstruction based on HR-MRI**

Based on the enhanced HR-MRI sequences, the lumen was initially reconstructed using ITK-SNAP (version 3.6.0-RC1; http://www.itksnap.org). Firstly, according to the location of the aneurysm, a cuboid region containing the complete aneurysm and part of the parent artery was segmented as a region of interest (ROI) in the software. Then the ITK-SNAP Active Contour Segmentation Mode function was used for automatic reconstruction. This process is divided into three main steps. Step 1: Image intensity thresholds are determined. To reconstruct the aneurysm wall, the lower limit of the image intensity of the aneurysm wall is used; when reconstructing the lumen, the threshold is the upper limit of the image intensity of the lumen. Step 2: Place bubbles in the image to initialize the contours of the reconstructed model growth. By placing multiple bubbles and adjusting the bubble size, the speed and precision of the growing process can be accelerated. Step 3: Adjust the parameters of the difference equation used for model growth and perform the growth process. All parameters are set by default. After obtaining the preliminary reconstructed model outline, use Paintbrush Mode to fine-tune and finalize the model, adding or deleting areas that are missing or multi-selected during the automatic reconstruction process, generating the final segmentation result, as shown in **Figure 1**. The segmentation results are exported as STL format files. The high-resolution MRI model in this step is only used as a reference and is not involved in subsequent analysis.

**Lumen model generation based on DSA**

DSA with a voxel size of 0.2mm*0.2mm*0.2mm provides higher accuracy than MRI (0.4mm*0.4mm*0.5mm). Moreover, the MRI voxel size interferes with calculations. A 3D lumen model was obtained by segmenting 3D rotated DSA images via MIMICS (Mimics 10.0; Materialise, Leuven, Vlaams-Brabant, Belgium). The segmentation derives from the aneurysm and lumen size measured by 2D-DSA angiography, the reconstruction used the level set method (1), with the image intensity threshold adjusted in MIMICS, so that the size of the reconstructed 3D model was the same as the angiography. The resulting size differential is under 0.2mm, that is within two-pixels. The segmentation results are exported as STL format files.

**Wall acquisition model matching of DSA and HR-MRI models**

Modification of the STL model files was completed in Geomagic (Geomagic Inc., Morrisville, North Carolina). The model coordinates output by ITK-SNAP remained unchanged, using the best-fit alignment function to match the lumen model output by MIMICS with ITK-SNPA, and then perform smoothing, trimming and other operations to fine tune and finalize the lumen model (flow chart in **Figure 2**).

**Wall enhancement model reconstruction via image intensity reading**

Since the size of an image voxel is similar to the thickness of the vessel wall, image intensity is partially affected by the volume effect, and voxels near the inner and outer sides will have lower intensity than the vessel wall (2). Therefore, a 3D model representing the middle layer of the vessel wall is generated by shifting the lumen model outward by 0.3 mm, approximately one pixel length, along the direction of the model surface mesh. The image intensity of the aneurysm wall was read using a script developed based on VTK8.2.0. The vessel surface reconstructed by DSA is offset outward to obtain an intermediate layer of the vessel wall for subsequent intensity measurements and to avoid volume effects leading to low measurement values. The sampling point spacing is 0.2mm (< voxel size 0.4*0.4*0.5mm). The VTK program was used to combine the model and image, and then output the image intensity of the position of the model surface. First, the spatial coordinates of the mesh points of the middle layer model of the vessel wall were read, then the image intensity of the enhanced sequence corresponding with each mesh point is read to generate a vtp file containing geometric information and image intensity values. The image intensity of the *pituitary* in each case is also read and the relative value of the image intensity of the aneurysm wall is calculated to obtain the aneurysm wall enhancement index CRstalk (3).

Because the subsequent calculation of hemodynamic parameters is performed in the lumen model, the image intensity from the middle layer of the vessel wall is projected on the lumen surface by using the closest matching spatial point in order to permit quantitative analysis of hemodynamics and vessel wall enhancement based on coordinate points. Projection is achieved by selecting a point (A) on the lumen surface model, to which the nearest point (B) on the mid-layer of the vessel wall can be found, and assigning the image intensity of point B to point A (**Figure** 3).

**Hemodynamic simulation**

In this study, considering the rapid changes in hemodynamic parameters within the lumen and near the aneurysm wall, a fully unstructured mesh with a 3-layer boundary (~1 million polyhedral elements with a maximum element size of 0.2 mm) was generated using the Star-CCM+ meshing tool (CD-Adapco, Melville, NY, USA). These meshes are used to solve the Navier-Stokes equations governing flow via the Star-CCM+ CFD solver, with a second order accuracy scheme, where blood is modeled as an incompressible laminar Newtonian fluid with a density of 1056 kg/m3 and a viscosity of 0.0035 kg/m·s. A no-slip boundary condition is assumed for the vessel wall, and the velocity boundary condition for the inlet. The average volumetric inflow rate was 1.3ml/s (4), with a typical flow velocity waveform (5). The flow boundary condition is set at each outlet, based on the principle of minimum energy, to ensure that the flow rate of each outlet is proportional to the cube of its equivalent diameter (6). The pulsatile flow simulation is run for three cardiac cycles to ensure numeric stability, with the third cardiac cycle used for post-processing.

**Hemodynamic parameters**

Wall shear stress (WSS) and oscillatory shear index (OSI) are two major hemodynamic parameters affecting intracranial aneurysms, playing important roles in aneurysm formation, growth, and rupture (7, 8). WSS is the viscous friction force of blood flow in the direction tangential to the blood flow on the vascular surface. For the simulation of pulsatile flow, time-averaged WSS (TWSS, **Eq. 1**) refers to the integration of the WSS amplitude at each node throughout the cardiac cycle; the low shear stress area (LSA) is defined as the area below 10% of the mean WSS value of the parent artery. While OSI (**Eq. 2**) represents the degree of flow field disturbance and quantifies the degree of WSS direction change within a cardiac cycle, OSI is a dimensionless parameter (value range 0-0.5). High OSI is defined as the ratio of the area where OSI is above 0.3.

$TAWSS=\frac{1}{T}\int_{0}^{T} |{wss}_{i}|dt$ （Eq.1）

$OSI=\frac{1}{2}\{1-\frac{|\int_{0}^{T} {wss}_{i}dt|}{\int_{0}^{T} {|wss}_{i}dt|}\}$ （Eq.2）

**WSS_i_ is the instantaneous WSS vector value and T is the cardiac cycle.**

The low flow volume (LFV) calculation process: 1. Isolate the aneurysm location (A); 2. Calculate 30% of the normal vascular flow rate; 3. Screen out the area with lower than 30% of the normal flow rate (B); 4. Calculate the volume of this area and find the volume ratio for the entire aneurysm; 5. Repeat the above steps to calculate the volume of the area that is lower than 10%, 20% and 40% of the normal blood vessel flow rate, obtain volume ratios, and select the appropriate rate *by comparison*.

Wall shear stress gradient (WSSG) measures the change in WSS in the flow direction, calculated by taking the spatial derivative of WSS with respect to the flow direction distance. The time-averaged WSSG in the dome area is further averaged. RRT is a combination of WSS and OSI, reflecting the time of residence blood spends near the aneurysm wall (**Eq. 3**). Gradient oscillatory number (GON) is a hemodynamic indicator used to quantify the degree of oscillatory tension (**Eq. 4**).

$RRT=\frac{1}{（1-2\times OSI）\times WSS}=\frac{1}{\frac{1}{T}|\int_{0}^{T} {wss}_{i}dt|}$ （Eq.3）

$GON=1-\frac{\left| \int_{0}^{T} Gdt \right|}{\int_{0}^{T} \left| G \right|dt}\left( 0\leq GON\leq1 \right)$ （Eq.4）

WSSi is the instantaneous WSS vector value, T the cardiac period; G = (ðfp/ðp, ðfq/ðq) the derivative of WSSG.

**REFERENCES**

1. Antiga L, Piccinelli M, Botti L, Ene-Iordache B, Remuzzi A, Steinman DA. An image-based modeling framework for patient-specific computational hemodynamics. *Med Biol Eng Comput*. (2008) 46. doi: 10.1007/s11517-008-0420-1

2. Kleinloog R, Korkmaz E, Zwanenburg JJ, Kuijf HJ, Visser F, Blankena R, et al. Visualization of the aneurysm wall: a 7.0-tesla magnetic resonance imaging study. *Neurosurgery*. (2014) 75: 614-22, 622. doi: 10.1227/NEU.0000000000000559

3. Roa JA, Zanaty M, Osorno-Cruz C, Ishii D, Bathla G, Ortega-Gutierrez S, et al. Objective quantification of contrast enhancement of unruptured intracranial aneurysms: a high-resolution vessel wall imaging validation study. *J Neurosurg*. (2021) 134: 862-9. doi: 10.3171/2019.12.JNS192746

4. Fahrig R, Nikolov H, Fox AJ, Holdsworth DW. A three-dimensional cerebrovascular flow phantom. *Medical physics (Lancaster)*. (1999) 26: 1589-99. doi: 10.1118/1.598672

5. Ford MD, Alperin N, Lee SH, Holdsworth DW, Steinman DA. Characterization of volumetric flow rate waveforms in the normal internal carotid and vertebral arteries. *Physiol Meas*. (2005) 26: 477-88. doi: 10.1088/0967-3334/26/4/013

6. Murray CD. The physiological principle of minimum work applied to the angle of branching of arteries. *J Gen Physiol*. (1926) 9: 835-41. doi: 10.1085/jgp.9.6.835

7. Jiang Y, Lu G, Ge L, Huang L, Wan H, Wan J, et al. Rupture point hemodynamics of intracranial aneurysms: case report and literature review. *Annals of Vascular Surgery - Brief Reports and Innovations*. (2021) 1: 100022. doi: 10.1016/j.avsurg.2021.100022

8. Xiang J, Tutino VM, Snyder KV, Meng H. Cfd: computational fluid dynamics or confounding factor dissemination? The role of hemodynamics in intracranial aneurysm rupture risk assessment. *AJNR Am J Neuroradiol*. (2014) 35: 1849-57. doi: 10.3174/ajnr.A3710
