# Supplementary figures and images for: Wall enhancement predictive of abnormal hemodynamics and ischemia in vertebrobasilar non-saccular aneurysms: a pilot study
Source: Front Neurol. 2023 Jun 2;14:1108904. doi: 10.3389/fneur.2023.1108904 (PMC10272805; doi:10.3389/fneur.2023.1108904)

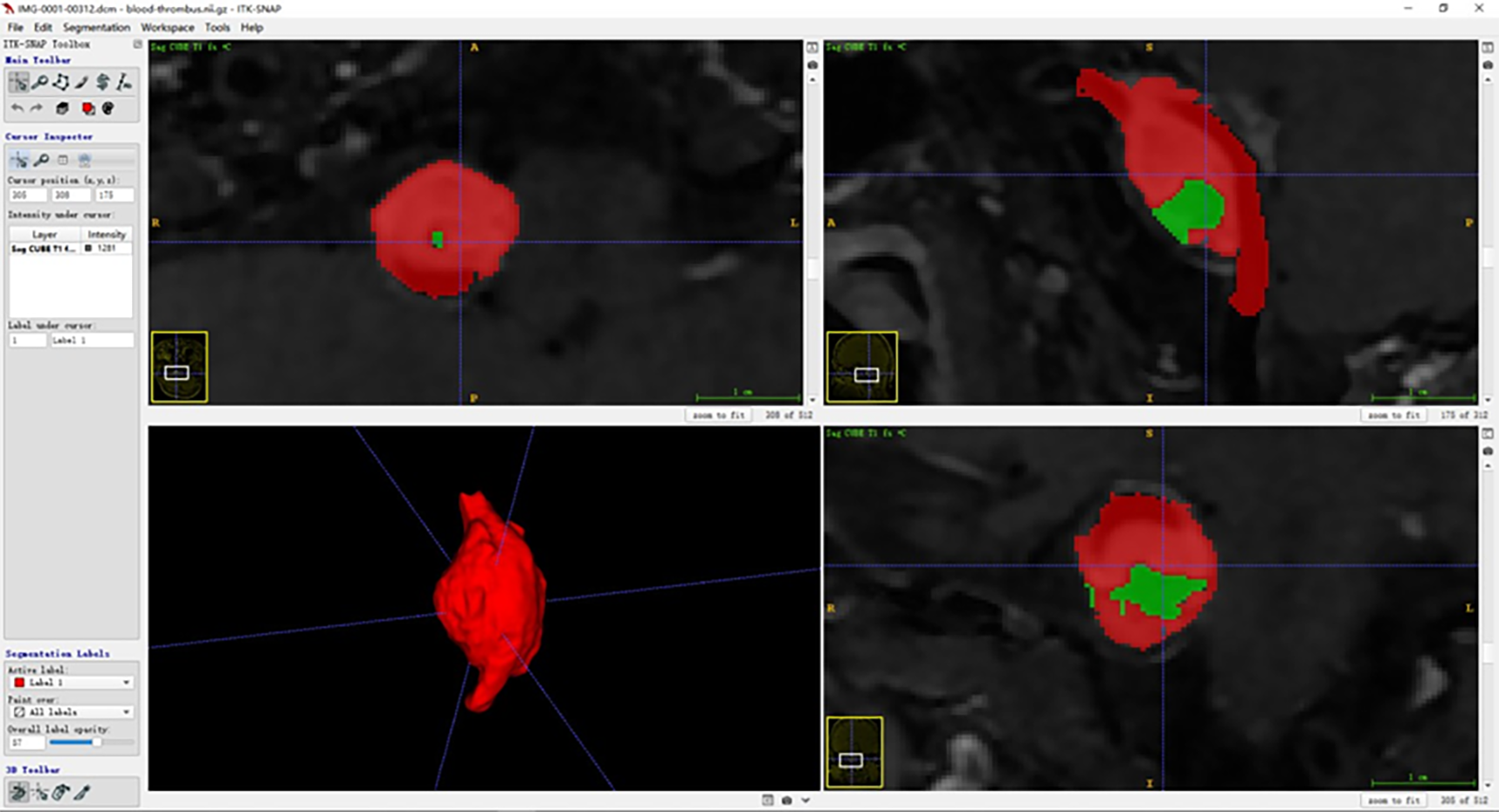

Supplement: Supplementary file 2 [file Image_1.PNG]

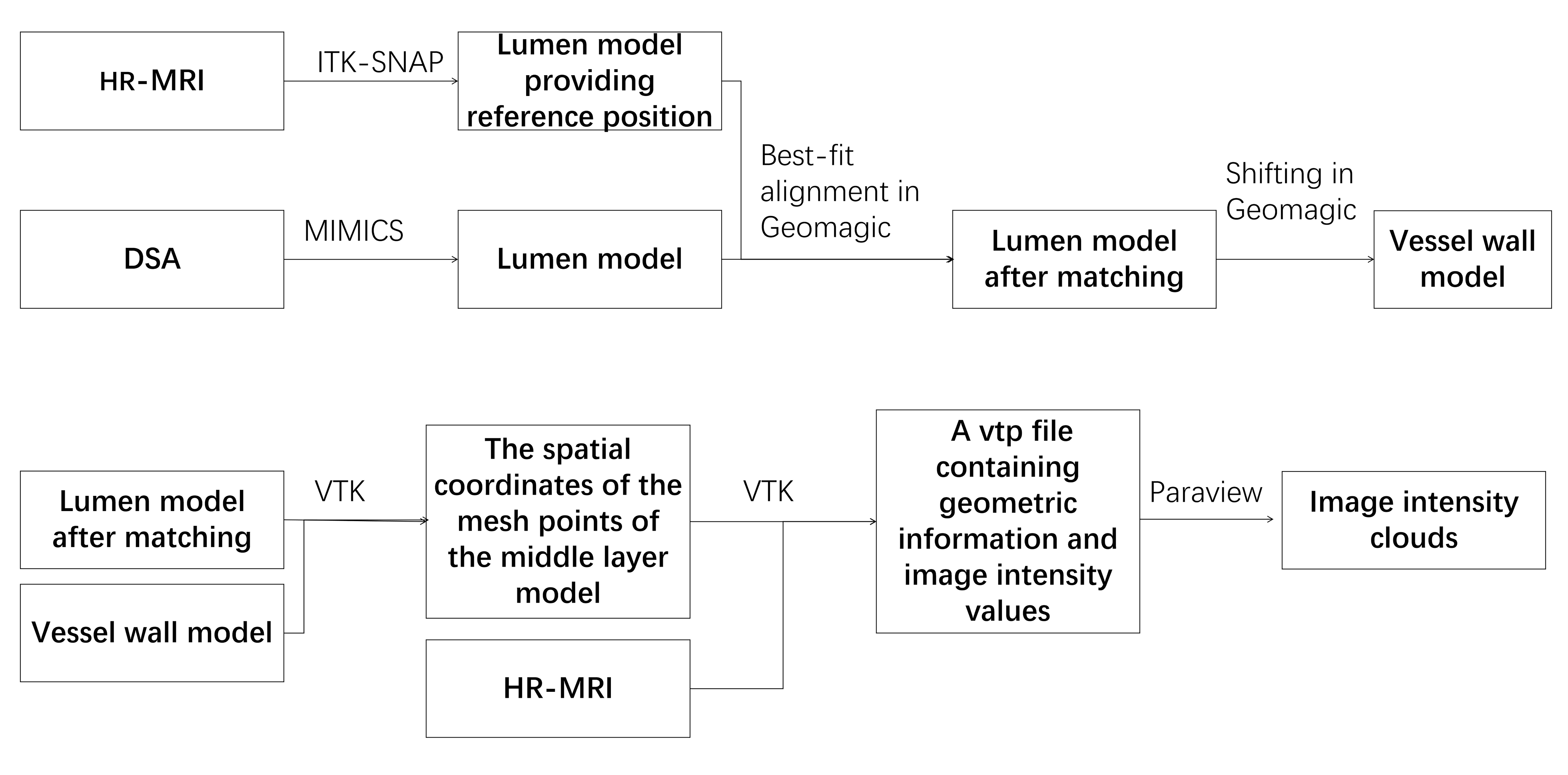

Supplement: Supplementary file 3 [file Image_2.PNG]

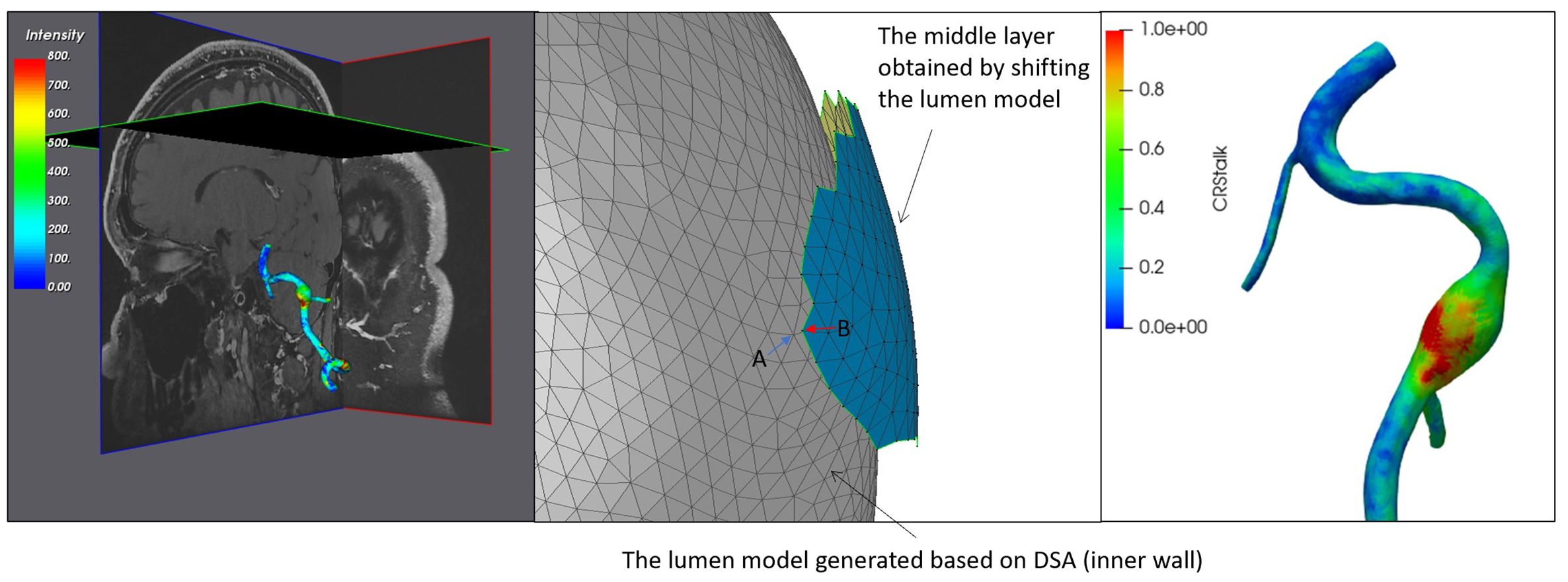

Supplement: Supplementary file 4 [file Image_3.jpg]
